# Intermittent theta burst stimulation vs. high-frequency repetitive transcranial magnetic stimulation for post-stroke cognitive impairment: Protocol of a pilot randomized controlled double-blind trial

**DOI:** 10.3389/fnins.2023.1121043

**Published:** 2023-03-30

**Authors:** Menglin Han, Jinyan He, Na Chen, Yulan Gao, Zhiqiang Wang, Kangling Wang

**Affiliations:** Department of Rehabilitation, Zhujiang Hospital, Southern Medical University, Guangzhou, China

**Keywords:** post-stroke cognitive impairment (PSCI), high-frequency repetitive transcranial magnetic stimulation (HF-rTMS), intermittent theta burst stimulation (iTBS), electroencephalography, randomized controlled trial

## Abstract

**Introduction:**

Intermittent theta burst stimulation (iTBS), a novel mode of transcranial magnetic stimulation (TMS), has curative effects on patients with post-stroke cognitive impairment (PSCI). However, whether iTBS will be more applicable in clinical use than conventional high-frequency repetitive transcranial magnetic stimulation (rTMS) is unknown. Our study aims to compare the difference in effect between iTBS and rTMS in treating PSCI based on a randomized controlled trial, as well as to determine its safety and tolerability, and to further explore the underlying neural mechanism.

**Methods:**

The study protocol is designed as a single-center, double-blind, randomized controlled trial. Forty patients with PSCI will be randomly assigned to two different TMS groups, one with iTBS and the other with 5 Hz rTMS. Neuropsychological evaluation, activities of daily living, and resting electroencephalography will be conducted before treatment, immediately post-treatment, and 1 month after iTBS/rTMS stimulation. The primary outcome is the change in the Montreal Cognitive Assessment Beijing Version (MoCA-BJ) score from baseline to the end of the intervention (D11). The secondary outcomes comprise changes in resting electroencephalogram (EEG) indexes from baseline to the end of the intervention (D11) as well as the Auditory Verbal Learning Test, the symbol digit modality test, the Digital Span Test findings, and the MoCA-BJ scores from baseline to endpoint (W6).

**Discussion:**

In this study, the effects of iTBS and rTMS will be evaluated using cognitive function scales in patients with PSCI as well as data from resting EEG, which allows for an in-depth exploration of underlying neural oscillations. In the future, these results may contribute to the application of iTBS for cognitive rehabilitation of patients with PSCI.

## 1. Introduction

Post-stroke cognitive impairment (PSCI), one of the most common complications of stroke (Zlokovic et al., 2020), refers to a variety of symptoms ranging from mild cognitive impairment to dementia. Approximately 20–70% of stroke survivors suffer cognitive impairment within 6 months of stroke (Liao et al., 2021; Merriman et al., 2021). Due to impaired attention, memory, language, and visuospatial functions, PSCI impedes recovery from stroke-related sequelae, including sensory impairment, motor dysfunction, and limitations in daily activities (Viktorisson et al., 2021). Currently, evidence-based treatment guidelines for PSCI are lacking, due to the limited pharmacological (donepezil, galantamine, and memantine) and non-pharmacological therapies (cognitive training and physical interventions) (Mijajloviæ et al., 2017). Hence, the identification of optimal and effective treatment is crucial. Recent studies have shown that neuromodulation techniques help improve cognitive impairment through neuroplasticity (Paolucci et al., 1996; Cicerone et al., 2011; Paolucci, 2013; Di Lazzaro et al., 2021), similar to repetitive transcranial magnetic stimulation (rTMS), which has been proven by several meta-analyses to have promising and positive effects (Lefaucheur et al., 2014, 2020; Hara et al., 2021; Liu et al., 2021; Zhang et al., 2021). Intermittent Theta Burst Stimulation (iTBS) is a novel neuromodulation technique with a unique advantage in treatment time compared to rTMS (Huang et al., 2005) and a reported better facilitation effect on modulating cortical excitability in brain regions (Blumberger et al., 2018; Kaster et al., 2019; Si et al., 2019). ITBS is effective and safe for treating depression, autism, and Parkinson’s disease in patients with mild cognitive impairment (Trung et al., 2019). However, evidence for iTBS treatment for patients with PSCI is limited. Some studies on PSCI have demonstrated that iTBS can improve patients’ overall cognitive function (Tsai et al., 2020; Li et al., 2022), specifically memory function (Tsai et al., 2020). However, the therapeutic mechanism of iTBS remains unclear, in the absence of sufficient neuroimaging assessments and long-term follow-up (Tsai et al., 2020; Li et al., 2022). In addition, there is inadequate evidence to suggest that iTBS is equally or more effective than traditional rTMS in terms of the treatment outcome. Therefore, we are conducting a randomized, double-blind controlled trial using neurobehavioral assessments combined with a neurobehavioral method, electroencephalogram (EEG), to compare the difference in effect between iTBS and rTMS in treating PSCI, as well as to explore neuroelectrophysiological changes. We hope to provide a theoretical basis for PSCI treatment. The protocol for this trial has been prepared according to the recommendations for interventional trials (SPIRIT) 2013 guidelines (Chan et al., 2013).

## 2. Methods and analysis

### 2.1. Patients

#### 2.1.1. Study setting

This study will be conducted at Zhujiang Hospital, Southern Medical University (Guangzhou, China). Forty inpatients with PSCI in the rehabilitation medicine ward will be included between October 2022 and December 2023.

#### 2.1.2. Eligibility criteria

Researchers will screen patients based on the inclusion and exclusion criteria. Once participants are confirmed as eligible, they will sign an informed consent form. To maintain safety criteria, we will not enroll patients who are intolerant to TMS. Women who are pregnant, breastfeeding, or intend to have children in the near future will not be eligible for enrolment. In addition, we will not enroll patients who have participated in other clinical trials or have a history of epilepsy. If a patient who has had TMS treatment has not received TMS treatment in more than 3 months, we may consider enrolling them. Finally, by conducting a preliminary exploratory study to compare clinical efficacy between rTMS and iTBS therapy on patients with PSCI, we aim to analyze data within and between the two hemispheres (the healthy and the affected sides) for the dynamic changes of oscillations to mine additional information. In light of the above statistical analysis, we decide to exclude patients with bilateral lesions from this study.

##### 2.1.2.1. Inclusion criteria

• Age 18 to 80 years;

• Stroke patients meeting the diagnostic criteria established at the Fourth National Cerebrovascular Disease Academic Conference in 1995;

• Imaging evidence of stroke confirmed by computed tomography (CT) or magnetic resonance imaging (MRI);

• Montreal Cognitive Assessment Scale (MoCA) score ≤24;

• Cognitive impairment should occur within 12 months of the vascular event and last for at least 3 months;

• First-ever stroke;

• Right-handed;

• Normal cognitive function before stroke;

• No severe aphasia (screened by the Chinese Aphasia Battery) and capable of completing cognitive tests;

• Stable vital signs;

• Voluntary participation and signed informed consent (signed by the patient or another authorized representative).

##### 2.1.2.2. Exclusion criteria

• Complete damage to the left prefrontal cortex confirmed by CT/MRI;

• Bilateral brain lesions;

• Defect of the skull;

• Use of antidepressants or psychostimulants;

• Metal or cardiac pacemaker implants near the treatment site;

• Previous brain disorders such as brain tumors, brain trauma, and seizures;

• History of malignant trauma;

• Unstable vital signs or failure of vital organs;

• Any neuropsychiatric comorbidity or affective disorder that could affect the test results;

• Patients with dementia (Clinical Dementia Rating grade ≥0.5) who are unable to cooperate with the cognitive assessment and intervention described below.

#### 2.1.3. Participant timeline

**FIGURE 1:**
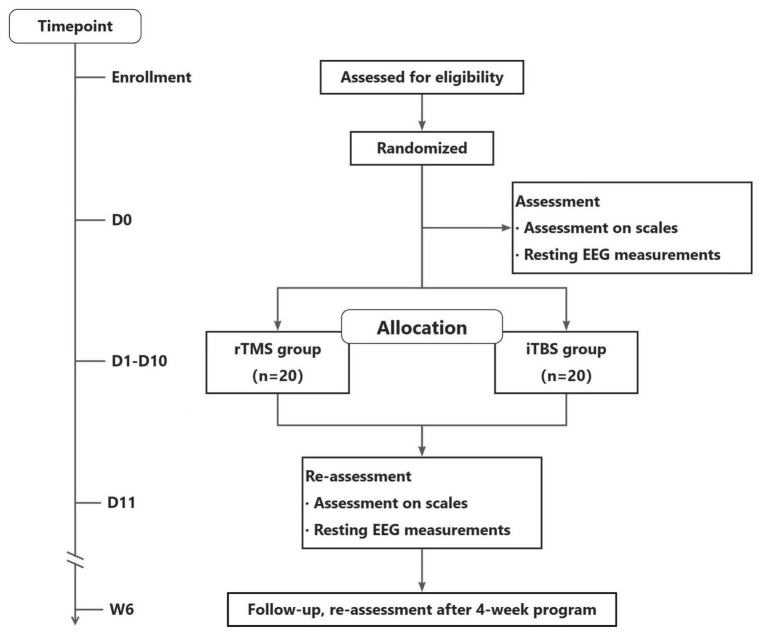
Design and flow of participants through the study.

The study will begin by screening the participants for eligibility. Once the patient’s eligibility has been confirmed, an informed consent form will be signed, and the patient will be randomly assigned to one of the two treatment groups. Clinical assessment and resting EEG measurements will be performed at baseline (D0), after 10 TMS treatments (D11), and at the 6-week follow-up (W6). It is possible that some participants will not be able to attend our hospital for their evaluation at W6. Therefore, we will present two situations. Participants who can return to the hospital will have their EEG data collected at W6, while those who are unable to return to the hospital will be evaluated door-to-door. A record will be made if a patient leaves the trial, is excluded, or withdraws at any point, along with the reasons. The visit schedule and study flowchart are presented in Table 1 and Figure 1.

**TABLE 1:** Trial schedule.

	Trial schedule				
	**Enrollment**	**Allocation**	**Post-allocation**	**Close-out**	**Follow-up**
**Timepoint**	**-D1**	**0**	**D1–D10**	**D11**	**W6**
**Enrolment**					
Eligibility screen	×				
Informed consent	×				
Allocation		×			
**Interventions**					
(rTMS)			×———×		
(iTBS)			×———×		
**Assessments**					
Demographics and clinical characteristics		×			
MoCA; AVLT; SDMT; DST; HAMD; ADL		×		×	×
Resting EEG		×		×	

DLPFC, dorsolateral prefrontal cortex; rTMS, repetitive transcranial magnetic stimulation; iTBS, intermittent theta burst transcranial magnetic stimulation; MoCA, Montreal cognitive assessment; AVLT, auditory verbal learning test; SDMT, symbol digit modalities test; DST, digital span test, HAMD: Hamilton depression scale; ADL, activities of daily living; EEG, electroencephalography.

#### 2.1.4. Sample size

This will be an exploratory study. The required sample size for this study was estimated using GPower software (version 3.1.9.7) (Faul et al., 2007). Repeated measures analysis of variance (ANOVA) will be used for statistical analysis, with group and time as affecting factors. Accordingly, the *F*-test (repeated-measures ANOVA, between factors) was chosen, with a power of 85%, an alpha value of 0.05, and an effect size of 0.25 (Cunningham and Mccrum-Gardner, 2007). The predicted minimum sample size was 32 (two groups) considering a 20% loss to follow-up. Therefore, we set the sample size at 40 patients in total, with 20 patients in each group.

#### 2.1.5. Recruitment

Forty inpatients with PSCI at Zhujiang Hospital, Southern Medical University will be recruited. The participants will be screened by a dedicated individual for those who meet the inclusion and exclusion criteria and are willing to receive TMS treatment. The participants will receive information in both written and verbal formats about the purpose and procedures of the study once their verbal consent has been confirmed. In this study, no biological specimens will be collected for storage, and no severe adverse effects on the participants are expected (Lefaucheur et al., 2020). The study will begin with a baseline assessment followed by random allocation after written informed consent is obtained from the participants.

#### 2.1.6. Randomization and blinding

Using a random number sequence generated using SPSS 25.0 software, all eligible patients will be randomly assigned to one of two treatment groups. The allocation and detailed TMS protocol will be known only to the two doctors who will perform TMS stimulation but will be blinded to the patients and other members of the study staff (such as outcome assessors or data analysts). Doctors performing TMS interventions will not be involved in any other aspect of the study, such as patient recruitment, randomization, allocation, outcome assessment, or data analysis.

### 2.2. Interventions

Transcranial magnetic stimulation treatments will be delivered by a magnetic simulator (Magneuro100, VISHEE Medical Technology Co., LTD, Nanjing, China) with a figure-8 coil. Each patient will receive TMS stimulation in the afternoon for 10 consecutive days. The left DLPFC (F3) will be the target site to stimulate the left prefrontal cortex according to the international 10/20 EEG recording system (Kim et al., 2010). The intensity will be set at 80% of the resting motor threshold in both the rTMS and iTBS groups (Shajahan et al., 2002; Kim et al., 2010).

#### 2.2.1. rTMS protocol

The 5 Hz rTMS parameters include 2-s trains (10 pulses) at an intertrain interval of 8 s, repeated every 10 s for a total of 10 min and 600 pulses.

#### 2.2.2. iTBS protocol

The iTBS parameters include three continuous pulses at 50 Hz, repeated at 5 Hz (2 s on, 8 s off) for a total of 192 s and 600 pulses. After the stimulation is completed, the direction of the coil is turned 90°, and a sham stimulation lasting 408 s is conducted in order to ensure the consistency of treatment time such that all patients are blinded to the experimental protocol.

#### 2.2.3. RMT

The resting motor threshold (RMT) refers to the minimum stimulus intensity that can evoke a response at least 50% of the time in a given number of trials (usually 10 trials). The patients will be asked to relax with their eyes open. During the recording process, the coils will be systematically moved (mapped) over the primary motor cortex until the maximal consistent response of the contralateral first dorsal interosseous muscle is detected. The RMT between the first dorsal interosseous bone and the minimum intensity is defined as the ability to elicit motor-evoked potentials of at least 50 mV in 5 out of 10 consecutive treatments (Rossini et al., 2015).

#### 2.2.4. Routine medical care

Medical care based on the disease of each patient is permitted.

#### 2.2.5. Discontinuation criteria

Patients with worsening symptoms, such as recurrent stroke, decreased muscle strength, or persistent unexplained infections.

Patients who wish to discontinue participation.

Patients who are unable to complete the treatment sessions.

Patients who are unable to participate in the baseline assessment.

### 2.3. Outcomes

Our primary outcome will be the Montreal Cognitive Assessment Beijing Version (MoCA-BJ) score from baseline to the end of the intervention (D11). Secondary outcomes will be resting EEG indexes, the Auditory Verbal Learning Test (AVLT), the symbol digit modality test (SDMT), the Digital Span Test (DST), and adverse events. Indexes of resting EEG include the absolute power and relative power of neural oscillations. Other outcomes include the Hamilton Depression Scale (HAMD) and Activities of Daily Living (ADL). All clinical assessments will be performed thrice: pre-treatment (baseline), post-last treatment, and at the 1-month follow-up. An experienced physician will conduct all cognitive assessments throughout the study and will be blinded to the participants’ group assignment and trial phases. The resting EEG will be conducted at baseline and at the end of TMS treatment.

#### 2.3.1. MoCA-BJ

The MoCA-BJ is a Chinese version of the Montreal Cognitive Assessment that is highly sensitive and specific for screening cognitive impairment in stroke patients (Nasreddine et al., 2005; Yu et al., 2012).

#### 2.3.2. Resting EEG measurements

A resting EEG is a graphical representation of the spontaneous electrical activity of a population of brain cells. It is obtained by magnifying and recording spontaneous biopotentials of the brain from the scalp using sophisticated electronic equipment (Höller and Nardone, 2021). EEG signals will be recorded using an EEG cap equipped with 64 Ag/AgCl electrodes, arranged according to the Extended International 10–20 electrode placement system (Tamburro et al., 2020). A 5-min EEG recording will be conducted with participants seated comfortably in a sound-insulated, dimly lit room with their eyes closed. All channels will be referenced online to the bilateral mastoid and amplified using an amplifier (Compumedics Neuroscan, Neuroscan 8050). Data will be sampled at 2,048 Hz, with impedances kept below 10 kΩ for all channels throughout data collection.

The acquired EEG signals will be analyzed offline using MATLAB2013b. Given previous evidence that oscillatory dynamics are affected by rTMS (Thut et al., 2011; Veniero et al., 2011; Chota et al., 2021) and are closely related to cognitive improvement (Klimesch et al., 1993; Richard Clark et al., 2004; Iliopoulos et al., 2020), we will analyze the power spectrum and functional connectivity of each oscillation within and between hemispheres, between groups, and pre- and post-treatment. Oscillation ratios, such as θ/α ratio, θ/γ ratio, and (α + β)/(θ + δ) ratio will be further analyzed (Coben et al., 1985; Moretti et al., 2013; Sato et al., 2022).

#### 2.3.3. AVLT

In the neuropsychology literature, AVLT is frequently used to assess memory. The test measures immediate and delayed free recall, retroactive and proactive interference, and recognition through verbal learning (Hawkins et al., 2004).

#### 2.3.4. SDMT

Several cognitive operations require the evaluation of information processing speed, which can be achieved through SDMT (Silva et al., 2018).

#### 2.3.5. DST

The scale can be divided into digit span forward (DSF) and digit span backward (DSB), each of which consists of two sets of 2-digit to 10-digit tables. The total score of the DSF and DSB indicates the participant’s attentional functioning, with a higher score indicating better function (Jahanshahi et al., 2008).

#### 2.3.6. HAMD

Hamilton Depression Scale has been widely used in psychopharmacological and clinical research since the 1960s (Hamilton, 1967).

#### 2.3.7. ADL

The modified Barthel Index (MBI) is used to assess an individual’s ability to perform basic activities of daily living (Mahoney and Barthel, 1965; Shah et al., 1989). In this study, a Chinese version of the MBI that includes ten items (personal hygiene, bathing, feeding, toileting, stair climbing, dressing, bowel control, bladder control, walking or wheelchair transfers, and chair-bed transfers) will be used (Leung et al., 2007). Total independence is indicated by a score of 100.

#### 2.3.8. Adverse events

During the treatment period and within 1 h following each treatment, adverse events, such as headaches, scalp sensations or nociception, temporal and neck muscle pain, and seizures will be recorded.

### 2.4. Statistical analyses

In the case of quantitative data, we will calculate the mean, standard deviation, and confidence interval as well as the minimum, maximum, P25, P50, and P75, as needed. For count data, we will calculate the frequency distributions and corresponding percentages. For rank data, we will provide frequency distributions and percentages, as well as median and mean rankings. Qualitative data will be presented as the positive rate, positive number, and denominator number of cases. Data from MoCA-BJ, AVLT, SDMT, DST, HAMA, and ADL will be analyzed by repeated-measures ANOVA using SPSS software (version 25.0; IBM, Armonk, NY, USA). Repeated-measures ANOVA will be used to analyze the differences between time points and groups. The acquired EEG signals will be analyzed offline using MATLAB2013b. The EEGLAB toolbox (version 13.0.0b) will be used for EEG data preprocessing (Delorme and Makeig, 2004), followed by interest channel selection based on the average level of the two groups and the relative spectral energy extracted from each frequency band. Finally, we will use repeated-measures ANOVA to compare the differences between groups and the relative spectral energy at different times. Statistical significance will be set at *P* < 0.05.

## 3. Discussion

Transcranial magnetic stimulation modulates brain function through neural changes induced by magnetic pulses. Using pulsed magnetic fields, TMS regulates the action potentials of nerve cells by inducing current in the central nervous system. This approach can affect the metabolic and neurophysiological activities of the brain. In recent decades, TMS has been widely used to treat cognitive impairment caused by various neurological, psychiatric, and psychological disorders, including stroke, Alzheimer’s disease, Parkinson’s disease, and schizophrenia. While iTBS, a new model of TMS, has been proven to be effective in Parkinson’s disease with cognitive impairment, its use in patients with PSCI is unclear. Several studies have shown that rTMS is a safe and effective method for improving cognitive function (Chou et al., 2020; Jiang et al., 2020; Alyagon et al., 2021). Regarding neurophysiology, iTBS may have equal or greater excitatory effects than conventional TMS (Di Lazzaro et al., 2011; Bakker et al., 2015). However, previous evidence indicates that conventional high-frequency TMS facilitates neurogenesis in the motor cortex more effectively than iTBS in a rat model (Luo et al., 2017). Another study that applied rTMS to healthy individuals found that rTMS produced a greater response than iTBS (Curtin et al., 2017). In patients with PSCI, there is no consensus regarding whether conventional rTMS or iTBS is more effective.

To assess the effect of the treatment, neurobehavioral scales are often used in studies of PSCI (Tsai et al., 2020). However, scale results are sometimes, to some extent, subjective because of the evaluator’s judgment and the state of the patient (Tsai et al., 2020). Therefore, objective means of assessment are urgently needed. EEG is gaining increasing attention because it is a special and complex bioelectrical signal reflecting the functional state of the brain, with the advantages of high temporal resolution, non-invasiveness, and low cost. A direct effect of rTMS treatment on brain function is altered nerve oscillation, which can have a therapeutic effect by resetting the oscillations of the thalamus and cortex (Thut et al., 2011; Veniero et al., 2011; Chota et al., 2021). Rhythmic patterns of neural oscillations are believed to play a functional role in local processing and communication among neuronal systems (Fries, 2005; Thut et al., 2012). Different regions of the human cortex tend to oscillate at different frequencies. Thus, it is possible to study neural oscillation activity in more detail. Most cognitive processes are associated with a frequency band in the delta, theta, alpha, beta, or gamma range. Several researchers have suggested, based on high-quality correlative EEG data, that brain oscillations are involved in a variety of sensory and cognitive processes (Klimesch, 1999; Lu et al., 2022). However, a causal relationship can only be demonstrated by directly modulating the oscillatory signals. EEG is an effective and dependable tool for detecting neural oscillations in the brain. Thus, this study may be able to investigate the specific relationship between neural oscillations and TMS facilitative effects on cognitive function in more detail.

In conclusion, the goal of this study will be to compare effect differences among various TMS protocols (iTBS and conventional rTMS) on PSCI and analyze whether iTBS is non-inferior or superior to conventional rTMS treatment. Given that iTBS has a shorter treatment time, it is more convenient to use if its therapeutic effect is not inferior to that of rTMS. Thus, iTBS might prove more advantageous and convenient than the classic rTMS for outpatients. Furthermore, we hope to explore the neural activity changes underlying iTBS/rTMS intervention in PSCI and thus, provide a theoretical foundation for clinical applications.

This study has some limitations. It will be a single-center trial with a comparatively small sample size, due to recruitment difficulties associated with the management of coronavirus disease 2019. In addition, the heterogeneity of oral medication in patients with PSCI may also pose potential problems in measuring cortical excitability and therapeutic response (Cantone et al., 2020). Future multi-center studies should be conducted to mitigate these limitations.

## Ethics statement

The studies involving human participants were reviewed and approved by the Medical Ethics Committee of Zhujiang Hospital of Southern Medical University. The patients/participants provided their written informed consent to participate in this study.

## Author contributions

MH designed the study and drafted the manuscript. MH, JH, NC, YG, and ZW collected the clinical data. KW critically revised the manuscript and contributed the most important intellectual content. All authors have read and approved the final manuscript.
